# Normalized N50 assembly metric using gap-restricted co-linear chaining

**DOI:** 10.1186/1471-2105-13-255

**Published:** 2012-10-03

**Authors:** Veli Mäkinen, Leena Salmela, Johannes Ylinen

**Affiliations:** 1 Helsinki Institute for Information Technology HIIT, Department of Computer Science, University of Helsinki, P.O. Box 68 (Gustaf Hällstromin katu 2b), Helsinki, 00014, Finland

## Abstract

**Background:**

For the development of genome assembly tools, some comprehensive and efficiently computable validation measures are required to assess the quality of the assembly. The mostly used N50 measure summarizes the assembly results by the length of the scaffold (or contig) overlapping the midpoint of the length-order concatenation of scaffolds (contigs). Especially for scaffold assemblies it is non-trivial to combine a correctness measure to the N50 values, and the current methods for doing this are rather involved.

**Results:**

We propose a simple but rigorous *normalized* N50 assembly metric that combines N50 with such a correctness measure; assembly is split into as many parts as necessary to align each part to the reference. For scalability, we first compute maximal local approximate matches between scaffolds and reference in distributed manner, and then proceed with *co-linear chaining* to find a global alignment. Best alignment is removed from the scaffold and the process is iterated with the remaining scaffold content in order to split the scaffold into correctly aligning parts. The proposed normalized N50 metric is then the N50 value computed for the final correctly aligning parts. As a side result of independent interest, we show how to modify co-linear chaining to restrict gaps to produce a more sensible global alignment.

**Conclusions:**

We propose and implement a comprehensive and efficient approach to compute a metric that summarizes scaffold assembly correctness and length. Our implementation can be downloaded from http://www.cs.helsinki.fi/group/scaffold/normalizedN50/.

## Background

In *de novo* genome assembly (see e.g. [1]), the result is usually a set of strings called *scaffolds*. These are DNA strings containing runs of Ns denoting gap regions of estimated length. The parts separated by N-runs are *contigs*. A typical measure to assess how well the assembly has succeeded is N50 measure, which equals the length of the scaffold (or contig) overlapping the midpoint of the length-order concatenation of scaffolds (contigs). If there is a reference genome available (as in assembly challenges, see http://assemblathon.org[2] and http://gage.cbcb.umd.edu/[3]), one can align the scaffolds to reason about the accuracy. With contig assemblies one can report a *normalized* N50 value that takes into account only the parts of the assembly that can be aligned to the reference using standard local alignment tools. With scaffold assemblies, normalization is more complex, as local alignment results for the contigs need also to be chained to form global alignments for the scaffolds. This chaining is typically done using heuristic approaches. Only very recently more attention has been paid for this problem: In [2] they build multiple alignment for the scaffolds and reference, and represent it as an adjacency graph where there are edges for representing aligned contigs and for adjacencies proposed by scaffolds (and some other types, see [2] for details). Then one can look at maximal paths that alternate between these two types to form scaffold paths. All such maximal scaffold paths can be extracted and used in the computation of normalized N50 value (called *scaffold path NG50* in [2]). While this is a diligent approach given an adjacency graph, the overall approach is highly dependent on heuristics to compute the multiple alignment, which is a very challenging computational problem to be solved exactly. This is especially true now that the multiple alignment tool used needs to cope with rearrangements in order to be able to align the partly incorrect scaffolds correctly. Although there exists effective tools (see e.g. [4]) even for this hardest instance of multiple alignment, the question is if there is an alternative approach that can completely avoid hard computational subproblems.

We propose a much simpler but still rigorous approach to compute normalized N50 scaffold assembly metric that combines N50 with correctness measure; in principle, assembly is split into as many parts as necessary to align each part to the reference. For example, let reference be GTAAGGCGAGGCTGAGAGT and let the assembly consist of two scaffolds CTGNNNGT and AGAGTANNNNGAGG, with N50 being 14. If we split the assembly into CTGNNNGT, AGA, and GTANNNNGAGG, then each piece aligns perfectly and the normalized N50 is 8. We show that this process can be modeled by three well-defined subproblems, each of which has an efficient and exact algorithmic solution.

In more detail, one needs to allow mismatches and indels in the alignment so that only the real structural errors in the assembly are measured. Moreover, the gaps between contigs in a scaffold may not be accurate due to variation in insert sizes of the mate pair reads used for the scaffold assembly. Taking these aspects into account, it would be easy to construct a dynamic programming recurrence to find the best scoring alignment for a scaffold, allowing gaps (N-runs) to vary within given thresholds. However, the running time would be infeasible for any practical use; one iteration would take at least *O*(*mn*) time, where *m* is the total length of scaffolds and *n* the length of the reference.

We propose a practical scheme of computing an approximation of the normalized N50 metric using the common seed-based strategy: First compute all maximal local approximate matches between scaffolds and reference, then chain those local alignments that retain the order both in reference and in each scaffold. This approach is called *co-linear chaining*[5]. As it was originally developed for RNA-DNA alignment, there was no need for restricting gaps in the chains, as introns can be almost arbitrary long. For our purposes, DNA-DNA alignment, it makes sense to restrict the length of the gaps between consecutive local alignments, as gaps should not be much longer than the insert size of mate pair reads. Finally, this alignment is repeated extracting the largest correctly aligning part from each scaffold in each step. We note that our approach is rigorous in the sense that we can avoid heuristics in each of the three subproblems considered (see the discussion in the end).

In what follows, we assume that local alignments are given, and first concentrate on modifying co-linear chaining for the case of restricted gaps. Then we proceed in explaining our implementation of the normalized N50 computation incorporating the local alignment computation with gap-restricted co-linear chaining. We then give our results on an experiment demonstrating how normalized N50 can characterize good and bad scaffold assemblies. Discussion follows on other possible uses and variations of the method proposed.

## Methods

Let us assume that all local alignments between scaffold and reference genome have been computed, and we have a set of tuples *V *= {(*x*,*y*,*c*,*d*)} such that *T*[*x*,*y*] matches *P*[*c*,*d*], where *T*[1,*n*] is the genome and *P*[1,*m*] the scaffold. In *co-linear chaining* the goal is to find a sequence of tuples *S *=* s*_1_*s*_2_⋯*s*_*p *_∈* V*^*p*^ such that *s*_*j*_*.y *>* s*_*j*−1_*.y*, *s*_*j*_*.d *>* s*_*j*−1_*.d*, for all 1 ≤* j *≤* p*, and |{*i*|* i *∈ [*s*_*j*_*.cs*_*j*_*.d] *for some 1 ≤* j *≤* p*}| is maximized. That is, find tuples preserving order in both *T* and *P* such that the resulting *ordered* coverage of *P* is maximized. We review an efficient solution given in [5] and extend it for our purposes. First, sort tuples in *V * by the coordinate *y* into sequence *v*_1_*v*_2_⋯*v*_*N*_. Then, fill a table *C*[1…*N*] such that *C*[*j*] gives the maximum ordered coverage of *P*[1,*v*_*j*_*.d*] over the choice of any subset of tuples from {*v*_1_*v*_2_,…*v*_*j*_}. Hence max_*j*_*C*[*j*] gives the total maximum ordered coverage of *P*. Then one can derive the following formulae for *Cj*[5] depending on the case: (a) Either the previous tuple $v_{j^{\prime }}$ does not overlap *v*_*j*_ in *P*; or (b) the previous tuple $v_{j^{\prime }}$ overlaps *v*_*j *_in *P*. For (a) it holds

(1) $$C^{a}[j]=\underset{j^{\prime }:v_{j^{\prime }}.d<v_{j}.c}{\text{max}}C[j^{\prime }]+(v_{j}.d-v_{j}.c+1).$$

For (b) it holds

(2) $$C^{b}[j]=\underset{j^{\prime }:v_{j}.c\leq v_{j^{\prime }}.d\leq v_{j}.d}{\text{max}}C[j^{\prime }]+(v_{j}.d-v_{j^{\prime }}.d).$$

Then the final value is *C* [*j*] = max(*C*^a^ [*j*],*C*^b^ [*j*]). Now we can modify the formulae taking the invariant values out from the maximizations to obtain *range maximum queries*. These can be solved using a search tree T that supports in *O*(log*n*) time operations *Insert*(*v*, *i*) to add value *v* to the tree with key *i* (if key *i* is already in the tree, replace its value $v^{\prime }$ with max(*v*, *v*^*′*^)); *Delete*(*i*) for deleting node with key *i*; and *v *=* Max*(*l*,*r*) to return the maximum value *v* from nodes {*i*} that belong to the interval *l *≤* i *≤* r*. Since there are two different maximizations, we need to maintain two different search trees. Notice that applying the recurrence directly would yield a trivial *O*(*N*^2^) time algorithm, whereas the use of invariant and search tree gives *O*(*N *log* N*) time. The resulting pseudocode (analogous to one in [5]) is given below.

**Algorithm **CoLinearChaining(*V *sorted by*y*-coordinate: *v*_1_,*v*_2_,…,*v*_*N*_)

(1) T.Insert(0,0); I.Insert(0,0);

(2) **for ***j*←1** to*** N ***do**

(3) $C^{a}[j]\leftarrow (v_{j}.d-v_{j}.c+1)+T.\text{Max}(0,v_{j}.c-1)$;

(4) $C^{b}[j]\leftarrow v_{j}.d+I.\text{Max}(v_{j}.c,v_{j}.d)$;

(5) *C*[*j*]←max(*C*^a^[*j*],*C*^b^[*j*]);

(6) $T.\text{Insert}(C[j],v_{j}.d)$;

(7) $I.\text{Insert}(C[j]-v_{j}.d,v_{j}.d)$;

(8) **return **max_*j*_*C*[*j*];

The alignment given by applying the above algorithm allows arbitrary long gaps, which is not a desirable feature. The gaps between consecutive contigs in scaffolds are restricted by the mate pair insert size, which also tells that in a correct alignment to the genome the gaps should not deviate much from this value. It is easy to modify co-linear chaining to restrict gaps: Replacing $T.\text{Max}(0,v_{j}.c-1)$ with $T.\text{Max}(v_{j}.c-\text{maxgap},v_{j}.c-1)$ at line (3) in the pseudocode restricts the gap in the scaffold by maxgap. To obtain analogous effect simultaneously in the reference genome, is a bit more tricky. Let us first describe a method that works in the special case that *v*_*j*_*.y*−*v*_*j*_*.x *are equal for all *j* and then consider the modifications required to handle the general case. For the special case, one can deploy T.Delete() as follows: At step *j* of the algorithm, maintain the invariant that T only contains all tuples $v_{j^{\prime }}$ having $v_{j^{\prime }}.y\geq v_{j}.x-\text{maxgap}$ and *j*^*′ *^<* j*. This is accomplished by adding the following code between lines (2) and (3) and initializing *j*^*′ *^= 1: 

(3’) **while**$v_{j}.x-\text{maxgap}\leq v_{j^{\prime }}.y$**do**

(3”) $T.\text{Delete}(v_{j^{\prime }}.d)$; *j*^*′*^←*j*^*′*^ + 1

(For simplicity of exposition, this assumes values $v_{j^{\prime }}.d$ are unique keys. One can use e.g. tuples $\left(v_{j^{\prime }}.d,j^{\prime }\right)$ as keys to ensure uniqueness.) The correctness for the special case follows as *v*_*j*−1_*.x *≤* v*_*j*_*.x* for all *j *> 2, and one can thus delete values incrementally so that the invariant is satisfied. The method fails in the general case since we can have *v*_*j*−1_*.x *>* v*_*j*_*.x* and tuples with *y*-coordinate between [*v*_*j*_*.x*−maxgap,*v*_*j*−1_*.x*−maxgap] are deleted. To overcome this, one can modify the algorithm as follows. Duplicate tuples and use *x*-coordinate for one copy and *y*-coordinate for the other as the sorting key. Now each tuple has *left* and *right* occurrence in sorted *V *. Apply the above algorithm, but do deletions only on left occurrences. In addition, on left occurrences, compute *C*[*j*] with lines 3-5 in the algorithm above, add the pair (*v*_*j*_,*C*[*j*]) in a list of *active tuples*P instead of applying lines 6-7 above. On right occurrences, update *C*[*j*] again but before lines 6-7 above, take the maximum of that value and the one stored for active tuple *v*_*j*_ in P. Then remove *v*_*j*_ from P and recompute *C*[*j*^*′*^] for all active tuples $v_{j^{\prime }}$ in P choosing as *C*[*j*^*′*^] the maximum of its previous value and the value computed applying lines 3-5 in the algorithm above. The correctness now follows from the facts that (a) when *v*_*j*_ is added to the active tuples P, *C*[*j*] is the maximum value without overlapping tuples $v_{j^{\prime }}$ taken into account, and (b) all the overlapping tuples $v_{j^{\prime }}$ with $v_{j}.x\leq v_{j^{\prime }}.y<v_{j}.y$ have their right occurrence before that of *v*_*j*_ and hence trigger the update of active tuple (*v*_*j*_,*C*[*j*]).

## Results

We used swift[6] for producing local alignments: The program takes as parameters the minimum match length (minlen) and the maximum error level (maxerror) as a percentage determining that at most maxerror ×* L* edit errors can be in a match of length *L *≥ minlen. It then finds all maximal local alignments satisfying the parameter constraints. The process was distributed so that scaffolds were partitioned into equal size chunks and each chunk allocated to a different cluster node.

The rest of the process (co-linear chaining, extraction of alignments, computation of N50) was executed on a single machine. To compute the normalized N50 value, the process was hence to apply co-linear chaining iteratively, always extracting the best alignment and splitting the scaffold accordingly. The process was repeated until all pieces (that had a local alignment in the first place) found their matches. The N50 of the pieces obtained this way is then called the normalized N50. Reverse complements were taken account appropriately; scaffolds were aligned to both strands and only contig alignments with the same orientation were combined to form a scaffold alignment.

We have already used normalized N50 in [7] to compare different scaffolders. We report here an experiment that gives some more insight to the normalized N50 measure: We created a varying number of random intra-scaffold contig swaps to an assembly *MIP-elegans* in [7] and computed normalized N50 for each variant. This gives a sampling between good assembly and completely random assembly such that scaffold N50 stays the same in all versions, but accuracy of the assembly should drop. One can see from Table 1 that normalized N50 indeed reflects this expected behaviour. The percentages in Table 1 give the amount of contigs translocated. Coverage values are computed after the first iteration of co-linear chaining. The reference genome is *Caenorhabditis Elegans* of length 100.3 Mbp. The assembly was produced by the MIP Scaffolder of [7] and has N50 value 189704.

**Table 1:** Normalized N50 on original assembly versus varying randomized assemblies with the same N50

	**Original**	**10%**	**20%**	**30%**	**50%**	**100%**
Normalized N50	183891	92212	56964	43461	33533	30403
Genome coverage	0.9333	0.6410	0.4778	0.4153	0.3421	0.3311
Scaffold coverage	0.9859	0.6847	0.5071	0.4414	0.3642	0.3522

For the experiment we ran the validate_distributed.sh script of our tool with parameters maxerror 0.02, minlen 35, maxgap 5000 and numjobs 120. Here the two first parameters are the ones for swift explained earlier. Third is used for restricting gaps in co-linear chaining, and the last is for distributing the heaviest part of the computation (local alignments). The 120 swift jobs were distributed on 20 machines taking overall 115 minutes for one run. The rest of the computation took 3 minutes on a single machine.

## Discussion

The proposed method should also work for validating an RNA assembly against a DNA reference, by just setting the maximum gap length to the maximum possible intron length. Also one could use it for whole genome comparison between two species, by considering how many pieces one genome needs to be partitioned in order to align to the other. Such measure is not very accurate as it does not model a sequence of evolutionary events to explain the transformation, like the genome rearrangement distances, but the approach gives the number of breakpoints which can be used as a lower-bound. However, much more elaborate tools for that purpose have been developed [8].

**Figure 1:**
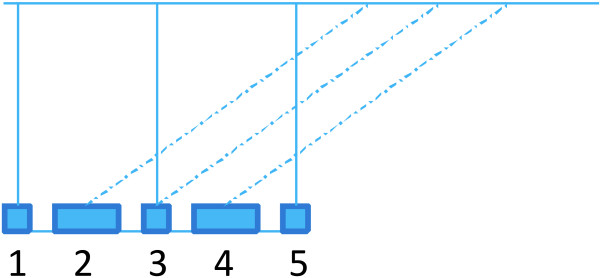
**An example where greedy extraction of gap-restricted co-linear chains may result into more pieces than optimal.** Greedy selection would align blocks 2, 3, 4 with dashed edges, but then with suitable gap-restriction blocks 1 and 5 could not be aligned together, and the assembly would be split into 3 parts. Optimal algorithm can choose 2 and 4 with dashed edges and then blocks 1, 3, 5 together, resulting into 2 parts only. It is possible to construct such an example even without multiple mappings for the blocks.

We stress that our approach has also some conceptual value in avoiding unnecessary heuristics. The three main steps (i) finding maximal local alignments, (ii) co-linear chaining, and (iii) splitting the scaffolds, have each an algorithmically correct solution. For (i) and (ii) one can refer to [5,6], as well as for the gap-restricted case covered in this paper. For (iii) one can refer to the folk theorem that greedy splitting of a string into maximal aligning pieces is optimal strategy if one wants to minimize the number of aligning pieces; this extends to the case of extracting aligning pieces from scaffolds greedily. It is interesting that actually with the gap-restricted co-linear chaining, this folk theorem does not hold anymore, see Figure 1. This leaves the open question whether there is an efficiently computable optimal strategy for splitting in this special case.

Finally, the approach in [2] is especially designed for evaluations where the reference consists of two haplotypes. Our approach is straightforward to modify for this case: The two haplotypes can be concatenated and used as the reference sequence to our program. This way the scaffolds will be split to parts that match one of the haplotypes only and the evaluation does not favor assemblies whose contigs or scaffolds alternate between haplotypes. On the other hand, obtaining assemblies that would separate the two haplotypes is quite unlikely with just short read sequencing data. It is also as easy to modify our approach for the case where haplotypes are allowed to mix: Assuming that the pair-wise alignment of haplotypes is known (which is the case with artificial data generated for evaluations), one can do the first step of our approach (maximal local alignments) separately for each haplotype, then project all the local alignment results to one haplotype using the known pair-wise alignment. After this the chaining allows haplotypes to mix.

## Conclusions

We proposed and implemented a comprehensive and efficient approach to compute a metric that summarizes scaffold assembly correctness and length. Our implementation can be downloaded from http://www.cs.helsinki.fi/group/scaffold/normalizedN50/.

## Competing interests

The authors declare that they have no competing interests.

## Authors’ contributions

VM and LS developed the gap-restricted version of co-linear chaining and it was implemented by VM. All authors contributed to the development of the normalized N50 framework and it was implemented and experimented by JY. All authors contributed to the writing. All authors read and approved the final manuscript.
